# Book Reviews

**Published:** 1898-03

**Authors:** 


### BOOK REVIEWS.

Lectures on Appendicitis, and Notes on Other Subjects. By Robert T. Morris, A.M., M.D., Fellow of the New York Academy of Medicine, American Association of Obstetricians and Gynecologists, American Medical Association, etc. Second edition, revised and enlarged. G. P. Putnam's Sons, 27 West Twenty-third street, New York. Price $2.00.

This little book is written in the same concise, dogmatical style as Dr. Morris's former work on "How We Treat Wounds To-day." It certainly has the "merit of brevity," and of setting forth the personal views of the author. Many important points, upon which the profession are laboring earnestly to reach satisfactory conclusions, the author has decided for himself, and dismisses with a wave of the hand. As a specimen of the author's style and dogmatism I quote the following:

"I speak, then, unequivocally, knowing that some patients are to die and others are to suffer unnecessarily because their advisers will believe themselves to be on a prognostic track. There is but one rule to be followed, and that is to isolate an infected appendix as promptly as we would isolate a case of diphtheria, and for practically the same reasons, viz.: the infected appendix will probably infect other structures, and the infected throat is likely to infect other throats. An infected appendix is isolated when it is out of the patient. All cases of appendicitis that are otherwise within surgical limitations, and that are within reach of competent surgical services, are cases for prompt isolation of the appendix. Various periods of waiting have been tried, with the effect of proving that the question is wedge-shaped, with the greatest number of deaths at the broad waiting end, and the smallest number of deaths at the point of isolating an appendix while infection is limited to the confines of the appendix. We are held to our rule by two cardinal principles, viz.: (1) Every hour of progress of any acute attack of appendicitis means increased damage to the viscera; and

(2) with no infected appendix the patient would have no complications of appendicitis, and therefore the patient would have no complications of appendicitis if we leave him with no infected appendix."

His statistics also are really quite amusing. The book contains many valuable practical suggestions, but, as a whole, would not, in the opinion of the reviewer, be a safe guide to follow, and must be taken *cum grano salis*. F. W. M.

---

Diseases of the Stomach. Their Special Pathology, Diagnosis, and Treatment, with Sections on Anatomy, Physiology, Analysis of Stomach Contents, Dietetics, Surgery of Stomach, etc. By John C. Hemmeter, M.D., Ph.D., Clinical Professor of Medicine Baltimore Medical College, etc. Illustrated. Pp. 788. $6.00. P. Blakiston, Son & Co., 1012 Walnut St., Philadelphia. [Or J. F. Meegan, Atlanta.]

This scientific and elaborate work, exhibiting thorough and painstaking research by the author, offers to the profession at large, and especially to the general practitioner, a volume of great worth, from which one can cull lessons of value both in the pathology and therapeutics of stomach and allied diseases. Beginning with the anatomy of the stomach, general and minute, the author next takes up the physiology of digestion and the phenomena of indigestion. He next considers bile, intestinal fermentation, putrefaction, organized ferments, the contemporaneous action of the several digestive secretions, methods of testing the motor functions of the stomach, method of testing gastric peristalsis, etc. Chapters 12 and 13 treat of stomach-washing, test-meals and the methods for qualitative and quantitative analysis of stomach contents.

Part second deals with the therapy and materia medica of stomach diseases. The first chapter discusses the principles of dietetic treatment of gastric disorders, and the second is devoted to the diet-kitchen and diet-lists. The latter contains valuable suggestions upon the proper mode of preparing foods, soups, fish, meats, jellies, vegetables, milk and egg dishes, etc. The third chapter discusses the dietetics of alcohol and alcoholic beverages. The author believes that alcohol is injurious to the human organism in health. Other chapters treat of the gastric douche and the uses and abuses of the natural mineral waters.

This is an up-to-date work in every particular, giving valuable information upon many heretofore obscure diseases and conditions of the stomach which have been overlooked or not fully investigated by writers on this subject. It is worthy of a place in every medical library. G. G. R.

---

SPINAL CARIES (Spondylitis or Pott's Disease of the Spinal Column). By Noble Smith, F.R.C.S., Ed., L.R.C.P., Lond., Surgeon to the City Orthopedic Hospital; Surgeon to All Saints' Children's Hospital; Orthopedic Surgeon to the British Home for Incurables. Second edition. Smith, Elder & Co., 15 Waterloo Place, London.

No better exhibition of the aims of this little book could be given than the preface to the second edition, which is quoted in full:

"In this edition I have corrected some errors, supplied an index, described a new form of head-piece for cervical disease, and have added some remarks upon the forced reduction of the deformity of caries under chloroform. I may add that since I wrote the first edition I have met with further confirmation of the conclusion which I then expressed, that spinal caries is generally a curable disease, but that success depends, above all things, upon accurate support of the spine. During this period very little has been done by surgeons generally to overcome the very unsatisfactory custom of leaving the construction of the apparatus entirely in the hands of instrument makers."

The author's method of treatment is at variance with the practice of the majority of orthopedic surgeons in America. They, as a rule, use plaster of Paris jackets and corsets, with jury masts where the disease is situated in the upper dorsal or cervical regions; while he uses braces of his own device.

The work of the publishers is well done, and the book contains many interesting and original illustrations. F. W. M.

TUBERCULOSIS OF THE GENITO-URINARY ORGANS, MALE AND FEMALE. By N. Senn, M.D., Ph.D., LL.D., Professor of Practice of Surgery and Clinical Surgery, Rush Medical College; Attending Surgeon to Presbyterian Hospital; Surgeon-in-Chief to St. Joseph's Hospital, Chicago. Illustrated. W. B. Saunders, 925 Walnut St., Philadelphia. Price $3.00.

Too much cannot be said in praise of this most excellent work on a very important and much neglected subject. Dr. Senn handles Tuberculosis of the Genito-urinary Organs in the same masterly manner that he has handled surgical tuberculosis of other organs and tissues. The whole field of literature of this subject has been thoroughly canvassed, cases cited, and the opinions of other pathologists and surgeons freely drawn from, making the book quite exhaustive, though without tiresome detail.

Tuberculosis of the male genitals is first considered, then tuberculosis of the female genitals, followed by tuberculosis of the bladder, urethra, and kidneys. The pathology of local tuberculosis is thoroughly given in Dr. Senn's most interesting style. Especially interesting and valuable, however, are the details of the most approved methods of treatment of these affections. Here also the author has drawn freely from his own cases, as well as quoted liberally. His further experience has confirmed his good opinion of the emulsion of iodoform in glycerine in local tuberculosis.

The work of the publishers has been well done, and the book is interesting from cover to cover. F. W. M.

---

A SYSTEM OF PRACTICAL MEDICINE. By American Authors. Edited by Alfred Lee Loomis, M.D., Late Professor of Pathology and Practical Medicine in the New York University, and William Gilman Thompson, M.D., Professor of Medicine in the New York University. Volume III. Per volume, Cloth, $5.00. Lea Brothers & Co., Publishers, Philadelphia and New York, 1898.

The subjects discussed in the third volume of this valuable system are: Diseases of the Alimentary Canal, Diseases of the Peritoneum, Diseases of the Liver and Gall-Bladder, Diseases of the Spleen, Diseases of the Pancreas, Diseases of the Thyroid Gland, Infectious Diseases Common to Man and Animals, Chronic

Metal Poisoning, Alcoholism, Morphinism, Purpura, Beri-Beri, Hemophilia, Diabetes, and Insolation. The authors assigned to these subjects are: Drs. R. C. Cabot, Boston; Warren Coleman, New York; George Dock, Ann Arbor; F. G. Finley, Montreal; J. E. Graham, Toronto; H. A. Hare, Philadelphia; W. B. James, New York; W. W. Johnston, Washington; A. A. Jones, Buffalo; F. P. Kinnicutt, New York; Alexander Lambert, New York; James Law, Ithaca; G. R. Lockwood, New York; H. M. Lyman, Chicago; W. F. McNutt, San Francisco; M. A. Starr, New York; James Stewart, Montreal; C. G. Stockton, Buffalo; and V. C. Vaughn, Ann Arbor.

The reader will find in this volume, as in its predecessors, the same thorough and scientific handling of subjects which will make this System when completed one of the most valuable that has been added to our literature in many years. The corps of contributors numbers nineteen able American authorities and teachers, and their articles may confidently be consulted for a complete statement of each subject in its practical bearings according to the most modern knowledge. Full therapeutic directions form a feature of this System and engravings and plates are introduced wherever they can aid in elucidating the text.

---

TEXT-BOOK ON SURGERY. General, Operative and Mechanical. By John A. Wyeth, M.D., Professor of Surgery in the New York Polyclinic Medical School and Hospital; late Surgeon to Mount Sinai Hospital, etc. Third Edition, Revised and Enlarged. D. Appleton & Co., New York. [Southern Agency, Atlanta.]

The preceding edition of this work, which was everywhere accepted as a standard text-book on surgery, was published in 1890. Since that time so many important advances have been made in surgical science and the operative technique that the author has found it necessary to revise and practically rewrite this volume. This edition exceeds the former by one hundred and twelve pages.

Many of our readers are familiar with Wyeth's Surgery. The author is a skilful surgeon, a good teacher and an accomplished writer. The last edition of his work, just from the press, may safely be assumed to be a complete exposition of modern surgery, and a careful examination of it will readily prove this. The chapters on Amputations, Fractures, Abdominal Surgery, Hernia, Ligation of Arteries, and Deformities are particularly full. Under Amputations sixty-nine cases of Wyeth's bloodless method of hip-joint amputation are tabulated, with the testimony of many of the operators as to the great value of this method. Mention should be made of the large, plain type and of the number and clearness of the illustrations. The present edition will still further enhance the popularity of this standard work.

---

A MANUAL OF OBSTETRICS. By A. F. A. King, M.D., Professor of Obstetrics and Diseases of Women in the Medical Department of the Columbian University, Washington, D. C. New (7th) edition. In one 12mo volume of 573 pages, with 223 illustrations. $2.50. Lea Brothers & Co., Publishers, Philadelphia and New York.

A work which is issued in a new edition every two years requires little more from the reviewer than the statement that it continues to deserve the reader's confidence and patronage by keeping closely in touch with advanced medical ideas. "In the present revision some errors and omissions in the preceding edition have been rectified; some additions introduced; the chapter on *Puerperal Septicemia* has been rewritten; and throughout the work an effort has been made to accentuate the importance of antiseptic midwifery with more emphasis than formerly."

This book has long enjoyed a well-deserved popularity, particularly with students. The essential principles of obstetrics have been nowhere more judiciously selected or more clearly presented.

---

CLINICAL DIAGNOSIS. By Dr. G. Klemperer, Professor at the University of Berlin. Translated by Nathan Brill, M.D., and Samuel Brickner, M.D., New York. The McMillan Company, 66 Fifth Ave., New York. Price $1.00.

As the name implies, this volume of 280 pages contains, in a very concise and well written manner, the "elements of clinical diagnosis." It contains the diagnosis of acute febrile, acute infectious diseases, diseases of the nervous and digestive systems, respiratory tract and circulatory system. For a volume of its size the practitioner will find it very useful for the study of clinical diagnosis. The chapters on the microscopic examination of the sputum and blood are especially interesting and instructive. Chapter 8, on the examination of the urine, contains the practical methods of urinary analysis. The chapter on the "diagnostic aids of the Roentgen rays" is a new addition to a work of this kind. The volume is nicely bound and well illustrated. W. L. C.

---

COLLIER'S WEEKLY has donned a dress entirely new and has almost doubled its quantity of illustrations. The current number is in larger type than its predecessors, and the three columns have been replaced by two broad ones. Five of the pages are entirely covered by illustations, besides which there are a dozen pictures; and among the artists represented are John La Farge, Frederic Remington, A. B. Wenzell, Eric Pape and Peter Newell. The first installment of Henry James's new novel, "The Turn of the Screw," appears, as well as the beginning of "An Impossible House Party"—an amusing serial by Caroline and Alice Duer. Another new contributor is Blanche Willis Howard, author of "One Summer," "Guenn," etc., who has a department entitled "Under the Sun." The new issue appears on heavy plated paper, and is in every way handsome and attractive.

---

The anesthetic is very often as much or more to be feared than the operation. This is especially so in the case of old persons and those who suffer from chronic or acute lung, heart or kidney disease. The greatest care should be taken that no more of the narcotic than is actually needed should be used. Oftener than is admitted, death from the anesthetic is due to lack of care or experience on the part of the anesthetist. When you are about to operate upon an individual who will probably take general narcosis badly, try local anesthesia. You will often be surprised at the apparently formidable operations which may be done with the aid of cocaine or eucaine.—*International Journal of Surgery.*

